# Medial Calcar Density Measured via Opportunistic Computed Tomography Is Well Represented by the Dorr C Classification

**DOI:** 10.7759/cureus.62428

**Published:** 2024-06-15

**Authors:** Rex W Lutz, Hope Thalody, Tia Alexander, Tyler Radack, Alvin Ong, Danielle Ponzio, Fabio Orozco, Zachary D Post

**Affiliations:** 1 Orthopedic Surgery, Jefferson Health New Jersey, Stratford, USA; 2 Orthopedic Surgery, Rothman Orthopaedic Institute, Egg Harbor Township, USA; 3 Orthopedic Surgery, Thomas Jefferson University Hospital, Philadelphia, USA; 4 Orthopedic Surgery, Orozco Orthopaedics, Linwood, USA

**Keywords:** hounsfield units, canal to calcar isthmus ratio, opportunistic computed tomography, dorr classification, total hip arthroplasty

## Abstract

Introduction

The canal-to-calcar isthmus (CC) ratio has been previously correlated with proximal femur osteology, but its relationship with bone density is not well established. Our purpose was to assess the relationship between femoral bone density, measured on opportunistic quantitative CT in Hounsfield units (HU), and CC ratio.

Methods

A total of 148 THA patients were included. The CC ratio was measured on anteroposterior hip radiographs. Using perioperative CT scans, a 1 cm diameter area was identified on a single mid-coronal slice in the medial calcar just proximal to the intertrochanteric ridge. The mean HU was calculated in this region to represent calcar bone density.

Results

Twenty-four percent (n = 35) of patients were classified as Dorr A (average CC ratio 0.47 [0.45; 0.48]), 67% (n = 96) as Dorr B (0.62 [0.55; 0.68]), and 11% (n = 17) as Dorr C (0.78 [0.77; 0.80]). There was a significant difference between Dorr A and Dorr C femurs (769 (144) vs. 588 (154) HU) as well as between B and C femurs (718 (166) vs. 588 (154) HU). The CC ratio was correlated with calcar bone density on CT (-0.370).

Conclusion

CC ratio is correlated with bone density determined by HU measurements on an opportunistic quantitative computed tomography scan, and bone density HU values were able to accurately differentiate bone density in Dorr A and B from Dorr C femurs. These findings suggest that the CC ratio is a reliable measurement to predict bone density in Dorr C femurs. Therefore, arthroplasty surgeons can confidently use the Dorr classification for patients with Dorr C femurs when preoperatively planning for THA.

## Introduction

Total hip arthroplasty (THA) is one of the most successful procedures in orthopedic surgery, and demand for THA continues to rise [1]. Fundamental principles of THA include optimizing femoral stem fixation, geometry, and positioning [2,3]. Historically, proximal femur osteology for THA was studied by Noble et al., who described the canal flare index. Similar to Noble et al., Dorr et al. provided a universal approach to measuring and recording proximal femur osteology from anteroposterior hip radiographs to assist THA surgeons preoperatively when determining the ideal femoral stem geometry for each patient [4,5]. Dorr et al. compared these radiographic measurements with bone histology and serum histochemistry and found that Dorr C femurs had significantly thinner cortices and a higher number of osteoblasts and osteoclasts, indicating higher bone turnover and osteopenia [5].

The most widely referenced measurement from Dorr et al. is the canal-to-calcar isthmus (CC) ratio, which has become shorthand for a patient’s proximal femur geometry and bone quality. In simple terms, the CC ratio is the ratio of the proximal femur isthmus to the proximal femur metaphysis [5]. This ratio can be divided into three groups: Dorr A (<0.5), Dorr B (0.5-0.75), and Dorr C (>0.75). Dorr A typically represents a femur with thick cortices and a narrow metaphysis and medullary canal, while Dorr C represents thin cortices with a wide metaphyseal and medullary canal. The authors recommended that Dorr A and B receive a non-cemented femoral component while Dorr C femurs receive a cemented component to minimize the risk for periprosthetic femur fractures while optimizing stem fit [5]. Recent trauma literature describing a series of 709 consecutive femoral neck fracture patients further illustrated that Dorr C proximal femurs are at an increased risk for periprosthetic femur fractures (15.9%) following cementless THA. Their data found no periprosthetic fractures in the cemented group, regardless of proximal femur osteology or Dorr classification [6]. According to the American Joint Replacement Registry (AJRR), cementless stem fixation remains the preferred fixation in primary THA in the United States. With that being said, the AJRR reports a significant reduction in early revision in cemented THA versus cementless THA in those over the age of 65. The report highlights the small but growing utilization of cemented stem fixation in primary THA [7].

The gold standard for measuring bone mineral density (BMD) is a dual-energy X-ray absorptiometry (DEXA) scan [8]. However, a DEXA scan can be difficult to obtain and is not typically available for preoperative THA planning due to logistical concerns and financial burdens [9]. Recent orthopedic literature has described the use of opportunistic quantitative computed tomography (qCT) scans to extrapolate bone quality from Hounsfield units (HU) [10-16]. This technology has been utilized to determine the risk of pedicle screw pullout [12]. Additionally, bone quality on opportunistic CT of the distal radius has been correlated with the risk of distal radius fractures [11]. Lastly, it has been used to predict native hip fracture risk in elderly patients [10]. Despite the well-documented use of qCT, there is a lack of information describing the use of this technology within the field of arthroplasty.

While the CC ratio has been correlated with proximal femur osteology, its relationship with bone density is not well established [5]. The purpose of this study was to correlate bone density (HU) measured within the medial femoral calcar to the CC ratio. We hypothesized that the bone density HU values of the proximal femur would correlate with the CC ratio. A well-established relationship between bone density and the CC ratio would allow surgeons to confidently rely on the readily available CC ratio to predict bone density during preoperative stem selection prior to THA.

## Materials and methods

Approval from the institutional review board of Atlanticare Regional Medical Center was obtained before the initiation of this study. A retrospective review was performed for 148 consecutive patients who underwent primary robotic THA by two fellowship-trained arthroplasty surgeons at our large academically affiliated private practice between January 2021 and December 2022. Race was self-reported by patients and included 121/148 (82%) individuals who were white, 6/148 (4%) were black, 1/148 (0.7%) were Asian, 2/148 (1.4%) were multiracial, and 18/148 (12.2%) denied reporting their race. All surgeries were primary THA, with indications for osteoarthritis in 145/148 (98%) of patients and avascular necrosis in 3/148 (2%) of patients. All femoral stems were cementless and included both a proximally coated, collarless, single-tapered, wedge stem in 71/148 (48%) of patients and a fully coated, collared, dual-tapered, fit-and-fill stem in 77/148 (52%) of patients. All THAs were performed with a direct anterior approach.

The inclusion criteria consisted of patients greater than 18 years old who underwent primary THA and had preoperative anteroposterior hip radiographs as well as preoperative CT scans of the ipsilateral hip available for review. Criteria for adequate pelvis radiographs included: the entire pelvis being able to be visualized from the iliac crest to the proximal femur; the obturator foramen and acetabular teardrops being symmetrical; the greater trochanters being in profile; and the lesser trochanters being partially superimposed over the femoral neck [17]. Patients with preoperative films not meeting these specific criteria were excluded. Each hip CT scan was obtained within eight weeks of the scheduled THA, according to the Stryker MAKO protocol for robotic THA planning [18]. The MAKO protocol specifies a CT scan performed with the patient lying supine with 1 mm cuts in the coronal, axial, and sagittal planes. All CTs included the operative hip extending 180 mm below the lesser trochanter. The CT energy was set to 140-160 kilovoltage, and the current was set to 200-250 milliamperes. All CT scanners were accredited by the American College of Radiology and passed both clinical and image-quality phantom testing prior to use [19].

**Figure 1:**
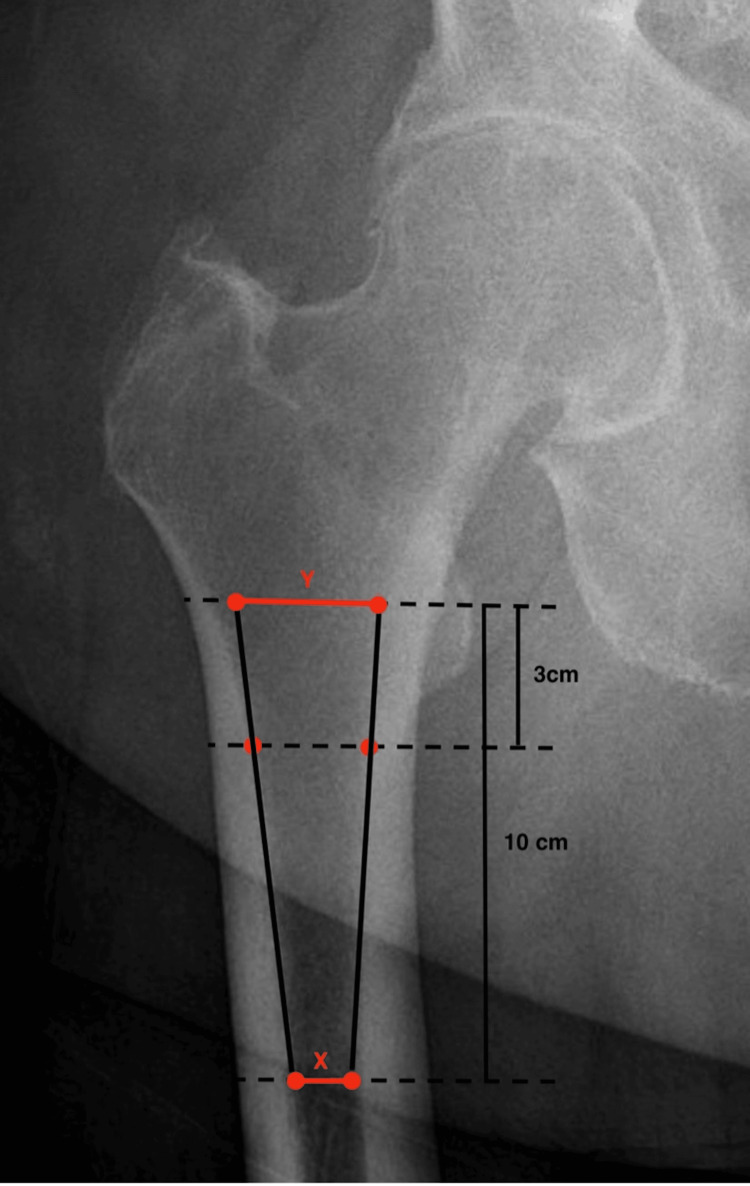
Schematic of the canal-to-calcar isthmus ratio The intramedullary width was measured 10 cm below the mid-lesser trochanter (X), representing the isthmus diameter. Two endosteal points were placed 3 cm below the mid-lesser trochanter. Two lines were placed tangential to the cortex, connecting the endosteal points at the 3 cm and 10 cm points. The width of these tangential lines was recorded at the level of the mid-lesser trochanter (Y), representing the metaphyseal diameter. The ratio of X/Y was recorded as the canal-to-calcar ratio.

A radiographic analysis was performed on each patient by two independent orthopedic surgeons who were not involved in the index THA. Radiographs were interpreted using our institution’s viewing software (Sectra Workstation IDS7, Sectra, Linkoping, Sweden) under 2× magnification. For each hip radiograph, the CC ratio was measured on the operative hip according to the method initially described by Dorr et al. [5]. First, the intramedullary width was measured 10 cm below the mid-lesser trochanter, representing the isthmus diameter. Two endosteal points were placed 3 cm below the mid-lesser trochanter. Two lines were placed tangential to the cortex, connecting the endosteal points at the 3 cm and 10 cm points. The width of these tangential lines was recorded at the level of the mid-lesser trochanter, representing the metaphyseal diameter. The isthmus-to-metaphyseal ratio (CC ratio) was recorded (Figure 1).

**Figure 2:**
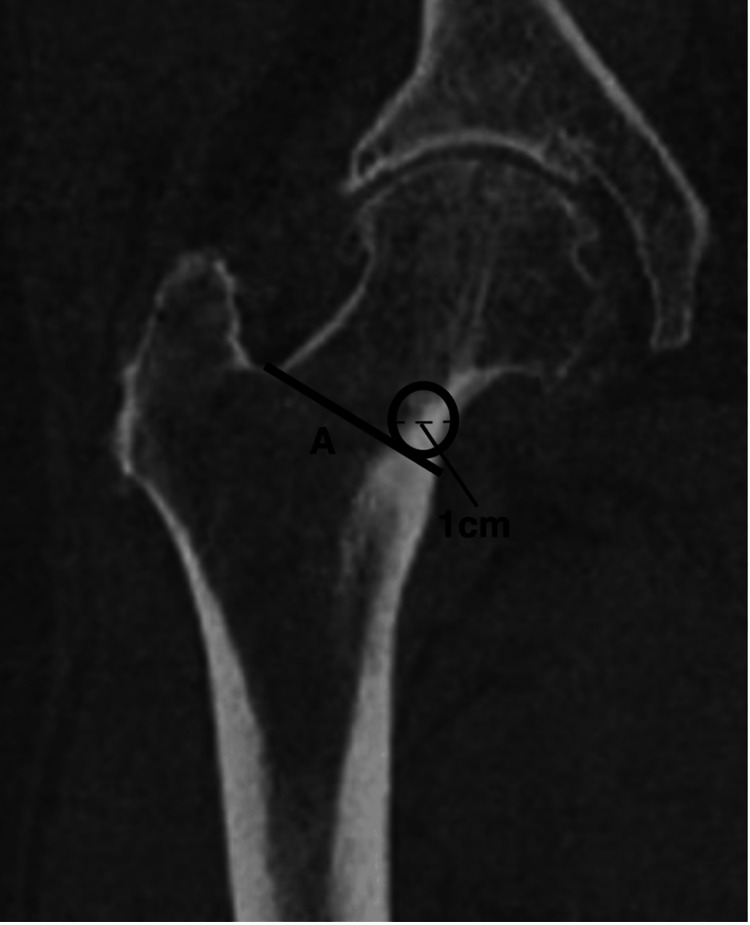
Schematic of qualitative computed tomography measurement A line was drawn along the intertrochanteric ridge on a mid-coronal slice of the hip computed tomography (A). A 1 cm diameter circle was drawn at the most medial aspect of the intertrochanteric line. This area was considered the region of interest and is where the Hounsfield units were measured.

BMD was determined on each CT scan by first finding the middle coronal slice between the most anterior and posterior femur slices on the coronal CT. A line was drawn along the intertrochanteric ridge as a reference point. A 1 cm diameter circle was placed along the most medial aspect of the cortex, just proximal to the intertrochanteric ridge and the lesser trochanter (Figure 2). The technique was modified based on the study performed by Pickhardt et al. to include the medial calcar [20]. This area was chosen because it was the area histologically sampled by Dorr et al. [5]. The HU was calculated in this region of interest via standard PACs software to represent the mean calcar bone density.

The final cohort was divided into three groups based on the Dorr classification. Patients were labeled as Dorr A if the CC ratio was <0.5, Dorr B if the CC ratio was 0.5-0.75, and Dorr C if the ratio was >0.75 [5]. The primary objective of our study was to correlate the mean calcar bone density measured with HU to the CC ratio. All statistical analyses were performed using R Studio Software (Version 3.5.1, Vienna, Austria). Normality was assessed via the Shapiro-Wilk test. Continuous data is presented as the mean (standard deviation) or median [first quartile; third quartile], and categorical data is presented as a percentage. Either ANOVA or Kruskal-Wallis tests were used to compare continuous data. Chi-square analyses were used for categorical data. Interobserver agreement was assessed for all radiographic measurements using agreement testing. Statistical significance was defined as P < 0.05.

## Results

Twenty-four percent (n = 35) of patients were classified as Dorr A (average CC ratio 0.47 [0.45; 0.48]), 67% (n = 96) were classified as Dorr B (average CC ratio 0.62 [0.55; 0.68]), and 11% (n = 17) were classified as Dorr C (average CC ratio 0.78 [0.77; 0.80]). There was a trend toward an increasing age from Dorr A to C; however, there was no significant difference in age between the groups. Furthermore, there was no difference in sex or laterality (Table 1). The interclass correlation between the two surgeons measuring the CC ratio was 0.866, demonstrating strong agreement and reliable measurements. There were no periprosthetic fractures within the study group.

**Table 1:** Demographics

Demographic	Total data	Dorr A	Dorr B	Dorr C	P-value
	N = 148	N = 35	N = 96	N = 17	
Age	69.0 [61.0; 75.0]	69.0 [61.5; 73.5]	68.0 [61.0; 74.0]	74.0 [65.0; 78.0]	0.177
Sex					0.196
Female	83 (56.1%)	19 (54.3%)	51 (53.1%)	13 (76.5%)	
Male	65 (43.9%)	16 (45.7%)	45 (46.9%)	4 (23.5%)	
Laterality					0.114
Left	75 (50.7%)	23 (65.7%)	45 (46.9%)	7 (41.2%)	
Right	73 (49.3%)	12 (34.3%)	51 (53.1%)	10 (58.8%)	

**Figure 3:**
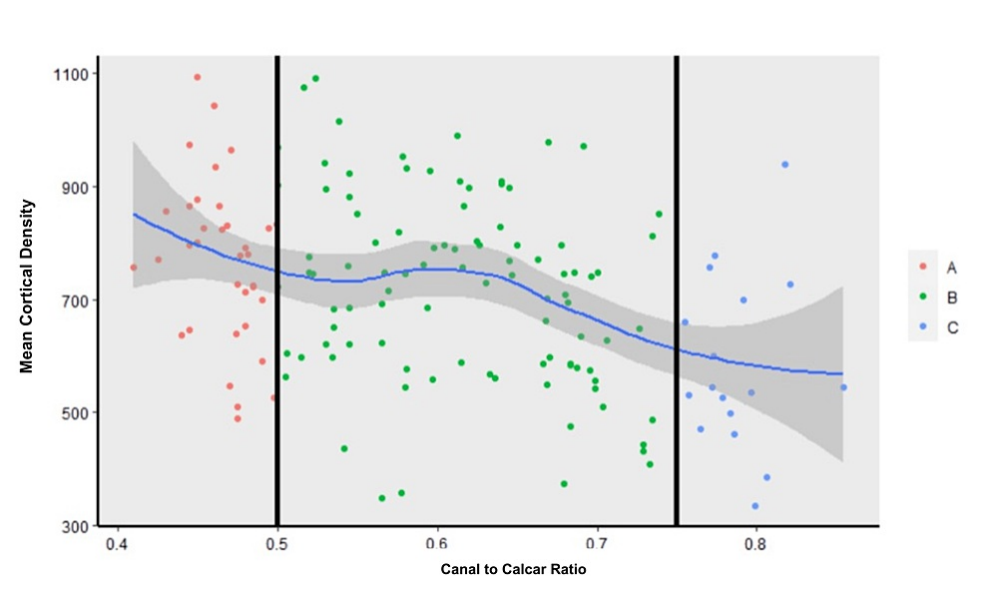
Mean cortical density vs. canal-to-calcar isthmus ratio This figure is divided into three sections according to the canal-to-calcar isthmus ratio. The far left (red) represents Dorr A (<0.5), the middle (green) represents Dorr B (0.5 to 0.75), and the far right (blue) represents Dorr C (>0.75). There is a correlation (-0.377) between mean cortical density and the canal-to-calcar isthmus ratio.

There was no significant difference in mean calcar bone density between Dorr A and B femurs (769 (144) vs. 718 (166) HU, p = 0.250). However, there was a significant difference between Dorr A and Dorr C femurs (769 (144) vs. 588 (154) HU, p = 0.001), as well as between B and C femurs (718 (166) vs. 588 (154) HU, p = 0.006) (Table 2). There was a negative correlation between the CC ratio and calcar density on CT (-0.370, Figure 3).

**Table 2:** Radiographic and computed tomography measurements

Measurement	Total data	Dorr A	Dorr B	Dorr C	P-value	P-value A vs. B	P-value A vs. C	P-value B vs. C
Average calcar-to-canal ratio	0.59 [0.50; 0.68]	0.47 [0.45; 0.48]	0.62 [0.55; 0.68]	0.78 [0.77; 0.80]	<0.001	<0.001	<0.001	<0.001
Cortical bone mean	715 (167)	769 (144)	718 (166)	588 (154)	0.001	0.250	0.001	0.006

## Discussion

The canal-to-calcar isthmus (CC) ratio, described by Dorr et al., has been correlated with proximal femur osteology and has been widely used to guide arthroplasty surgeons in determining the ideal femoral stem geometry and fixation in preparation for THA [5,6,21,22]. Surprisingly, and despite an abundance of surrounding literature, the CC ratio’s relationship with bone density is not well established.

There are several limitations to our study. Although we evaluated a large cohort of patients with CT data, there were only a small number of patients with Dorr C proximal femurs. Forty-eight patients were required in each Dorr group for statistical power, and for this reason, we are unable to calculate an exact range for which we recommend cement fixation, and we only provide an estimate. Additionally, this is a newly established protocol. We attempted to create a protocol based on a culmination of previously described literature to suit the needs of the arthroplasty surgeon. Further research is warranted to validate and reproduce our findings; however, this study does promote a novel technique that allows for reproducibility.

Our distribution of patients by Dorr classification (24% A, 67% B, 11% C) aligns with the study by Kheir et al., who found a Dorr A, B, and C classification prevalence of 21.1%, 66.3%, and 12.6%, respectively [6]. In contrast, Dorr et al. found a prevalence of 36.5%, 32.7%, and 30.8% of Dorr A, B, and C femurs, respectively [5]. Regardless, we demonstrated strong interobserver agreement between two independent reviewers and feel that our consecutive series is an accurate representation of our THA population.

Several studies have validated the correlation of HU with bone density [12,13,15,16]. Wang et al. utilized three standardized spine models with known densities and evaluated the associated qCT measurements, and the authors found that the measurements of bone density on qCT were both accurate and precise [16]. Brett et al. mentioned that qCT presents a unique opportunity for the healthcare system to diagnose and treat osteoporosis [15]. Anderson et al. echoed these thoughts and stated that qCT should be evaluated in patients who otherwise have not undergone a DEXA scan as an opportunity to screen for osteoporosis [12]. In their study comparing qCT to DEXA scans in the same patient, Khoo et al. demonstrated excellent correlation [13]. Given the availability of CT images during robotic THA and the unique relationship that qCT has with bone density, we found a unique opportunity to compare the CC ratio to the medial femoral calcar HU.

While many variations of qCT methodology exist, the protocol used in this study was developed to be easily reproducible by orthopedic surgeons with standard CT scans and PACs software. The region of interest tool allows the user to calculate the average tissue density, as represented in HU, in any area visible on a CT scan. It is important to note that our machines were all calibrated by the American College of Radiology and passed both clinical and image quality phantom testing [19]. Variation may occur in machines calibrated to different specifications or phantoms.

We found a correlation between HU and the CC ratio. Our findings demonstrate that Dorr A femurs have a higher medial calcar bone density, and Dorr C femurs have a lower medial calcar bone density. While there was a significant difference in the CC ratio between all Dorr cohorts, there was only a significant difference in qCT measurements between Dorr A and C and Dorr B and C cohorts. This information further validates the necessity for different treatments of Dorr A and B femurs (cementless stems) versus Dorr C femurs (cemented stems) as originally described [5]. Likely, Kheir et al. saw such a drastic difference in periprosthetic fractures between Dorr A and B femurs versus Dorr C femurs because of the large and significant difference in medial calcar density demonstrated in our analysis [6]. While the Dorr classification may be a shorthand method for geometric stem selection, we have demonstrated that bone density using a qCT does correlate with the CC ratio and, by extension, allows the CC ratio to provide more information on stem selection. Future studies should help expand or narrow the indications for cemented stem selection using qCT.

We discovered an average bone density of 588 (154) HU in the patients with a CC ratio of >0.75 (Dorr C). There are many unique risks and complications that arise for patients with decreased BMD who undergo THA or hemiarthroplasty [4,6,21]. Aro et al. demonstrated that patients with decreased BMD on DEXA scans had delays in osseointegration of their cementless THA. In addition, low BMD was associated with a higher subsidence and stem translation rate. Furthermore, a low canal flare index, as described by Noble et al., was associated with rotation instability [4,21]. Kheir et al. demonstrated Dorr C proximal femurs are significantly more prone to THA periprosthetic fractures when femoral neck fractures are treated with a cementless femoral stem, with a cementless THA periprosthetic fracture rate of 2.3%, 3.7%, and 15.9% in Dorr A, B. and C femurs, respectively. Therefore, they recommend a cemented femoral stem for Dorr C femurs [6].

We demonstrated a significant difference between Dorr A and Cand B and C femurs and their medial cortical density measured with HU on CT. Dorr C femurs had an average medial cortical density of 588 (154) HU. Thus, by taking into consideration data from prior studies, we can extrapolate that THA patients with a mean medial cortical density near 588 HU on opportunistic qCT scans should be considered for cement fixation of the femoral component. The only caveat is that the studies all analyzed THA for hip fractures rather than primary THA.

## Conclusions

We demonstrated that mean calcar bone density (HU) measurements on opportunistic qCT have a correlation with the CC ratio. Specifically, bone density HU values were able to accurately differentiate Dorr A and B from Dorr C femurs. These findings suggest that the CC ratio is an adequate measurement to predict bone density in patients classified as Dorr C. With these findings, arthroplasty surgeons can more confidently use the Dorr classification for Dorr C patients when making decisions regarding femoral stem geometry, size, and fixation methods.
